# Growth dynamics in *Acropora cervicornis* and* A. prolifera* in southwest Puerto Rico

**DOI:** 10.7717/peerj.8435

**Published:** 2020-02-11

**Authors:** Ernesto Weil, Nicholas M. Hammerman, Rebecca L. Becicka, Juan Jose Cruz-Motta

**Affiliations:** 1 Marine Sciences, University of Puerto Rico, Mayaguez, Puerto Rico, USA; 2 Gehrmann Laboratories, University of Queensland, St Lucia, Australia

**Keywords:** *Acropora cervicornis*, *A. prolifera*, Natural recovery, Growth and mortality, Puerto Rico

## Abstract

Natural population recovery of *Acropora palmata, A. cervicornis* and their hybrid, *Acropora prolifera*, have fluctuated significantly after their Caribbean-wide, disease-induced mass mortality in the early 1980s. Even though significant recovery has been observed in a few localities, recurrent disease outbreaks, bleaching, storm damage, local environmental deterioration, algae smothering, predation, low sexual recruitment and low survivorship have affected the expected, quick recovery of these weedy species. In this study, the status of three recovering populations of *A. cervicornis* and two of *A. prolifera* were assessed over one year using coral growth and mortality metrics, and changes in their associated algae and fish/invertebrate communities in three localities in the La Parguera Natural Reserve (LPNR), southwest coast of Puerto Rico. Five branches were tagged in each of 29, medium size (1–2 m in diameter) *A. cervicornis* and 18 *A. prolifera* colonies in the Media Luna, Mario and San Cristobal reefs off LPNR. Branches were measured monthly, together with observations to evaluate associated disease(s), algae accumulation and predation. *A. cervicornis* grew faster [3.1 ± 0.44 cm/month (= 37.2 cm/y)] compared to *A. prolifera* [2.6 ± 0.41 cm/month (= 31.2 cm/y)], and growth was significantly higher during Winter-Spring compared to Summer-Fall for both taxa (3.5 ± 0.58 vs. 0.53 ± 0.15 cm/month in *A. cervicornis,* and 2.43 ± 0.71 vs. 0.27 ± 0.20 cm/month in *A. prolifera*, respectively). Algal accumulation was only observed in *A. cervicornis,* and was higher during Spring-Summer compared to Fall-Winter (6.1 ± 0.91 cm/month and 3.8 ± 0.29 cm/month, respectively, (PERMANOVA, df = 2, MS = 10.2, *p* = 0.37)). Mortality associated with white band disease, algae smothering and fish/invertebrate predation was also higher in *A. cervicornis* and varied among colonies within sites, across sites and across season. The balance between tissue grow and mortality determines if colonies survive. This balance seems to be pushed to the high mortality side often by increasing frequency of high thermal anomalies, inducing bleaching and disease outbreaks and other factors, which have historically impacted the natural recovery of these taxa in the La Parguera Natural Reserve in Puerto Rico and possibly other areas in the region. Overall, results indicate variability in both growth and mortality rates in both taxa across localities and seasons, with *A. cervicornis* showing overall higher mortalities compared to *A. prolifera*.

## Introduction

Coral reefs are the most diverse and complex marine habitats on the planet and provide major ecological services to other important coastal and oceanic communities, and to human beings (Naeem, 1998; Huntington et al., 2017). However, the integrity of the community has been compromised by rising sea water temperatures linked to global warming (GW), which continue to affect both the periodicity and intensity of coral bleaching and disease outbreaks (Weil, Smith & Gil-Agudelo, 2006; Harvell et al., 2009; Hughes et al., 2018), as well as the frequency and intensity of storms, and changes in rain and drought patterns (IPCC, 2014). The combined impact of these stressors has produced drastic biological, ecological and structural consequences, leading to significant declines in live coral, community shifts and the collapse of the three-dimensional (acroporids) structure in many Caribbean and Indo-Pacific coral reefs (Aronson & Precht, 2001; Weil, Croquer & Urreiztieta, 2009; Hughes et al., 2018). In the Caribbean region, a mostly disease induced (white band disease) mass-mortality of *Acropora palmata* and *A. cervicornis* started in the late 1970’s, wiping-out over 90% of these species throughout the region (Gladfelter, 1982; Knowlton, Lang & Keller, 1990; Aronson & Precht, 2001). Populations were reduced to a few surviving colonies or fragments that in many localities had to compete with encroaching algae due to a lack of herbivory because of overfishing and the mass-mortality of the black sea urchin *Diadema antillarum* (Lessios, Robertson & Cubit, 1984). Soon after the mortality, the tri-dimensional, complex structure collapsed, significantly reducing essential fish habitat and niche availability for other invertebrate species, which impacted biodiversity and trophic dynamics in shallow Caribbean coral reefs (Knowlton, 1992; Weil et al., 2002; Acropora Biological Review Team, 2005).

Even though *Acropora cervicornis* and the hybrid *A. prolifera* are the fastest growing coral species in the Caribbean, populations have failed to recover region-wide, 35 years after the disease-induced mass mortalities (Precht et al., 2002; Mumby et al., 2006), with only a few populations now covering large areas (Vargas-Ángel, Thomas & Hoke, 2003; Keck et al., 2005; Busch et al., 2016; D’Antonio, Gilliam & Walker, 2016). New colonies and medium size thickets are generally transient, succumbing to recurrent WBD, bleaching, storms, predation, algae overgrowth and/or local habitat deterioration (Aronson & Precht, 2001; Precht et al., 2010; Weil & Rogers, 2011; Miller et al., 2014; Goergen et al., 2019). Low population levels and a lack of significant recovery for roughly 30 years, led to *A. palmata* and *A. cervicornis* being listed as critically endangered under the IUCN Red List of Threatened Species (Aronson et al., 2008).

Many strategies have been implemented to foster new *Acropora* growth, but success is highly variable and generally site-specific. Restoration efforts have aimed to supplement *Acropora* population recovery with locally cultured, fragment-propagation activities (Young, Schopmeyer & Lirman, 2012). Although survivorship of transplanted corals is relatively high after a year or two, similar to natural populations, studies show high mortality in subsequent years due to a major stressor or to one of the synergistic effects mentioned above (Quinn & Kojis, 2006; Garrison & Ward, 2012). In the absence of thermal stress, the major stressors seem to be (in order of increasing importance): damselfish gardening behavior, algal smothering, predation by invertebrates (i.e., snails and fire-worms) and recurrent but low prevalence of WBD-like problems (Wilkes et al., 2008; Weil & Rogers, 2011; Goergen et al., 2019). However, evidence presented in those studies on the overall functional roles of damselfish and their relationship with the host coral, has been variable and sometimes contradictory.

Regarding the specific mechanisms regulating damselfish-*Acropora* interactions, populations of damselfish have exploded in many localities due to the interactive effect of two processes: (1) lack of predators due to overfishing, and (2) availability of the newly created three-dimensional structure associated with the re-growth of acroporids, which provides refuge and habitat to the juveniles and then, the adults (Cole, Pratchett & Jones, 2008; Huntington et al., 2017). It has been consistently shown that damselfishes rapidly colonize young and older acroporid colonies, and actively bite and kill polyps to establish their algae lawns and nesting territories (Potts, 1977; Suefuji & Woesik, 2001; Wilkes et al., 2008). The bite injuries and further tissue mortality facilitates macro-algae growth and accumulation, as well as inducing focal infections of white band disease (Weil et al., 2002; Weil et al., 2002; Weil & Rogers, 2011).

Recent significant recovery of several back reef populations of *A. cervicornis* and the F1 hybrid *A. prolifera* were observed and investigated for over three years in two reef localities in southwest Puerto Rico (Lucas & Weil, 2015). This has provided a rare opportunity to evaluate some of the interactions proposed in the literature related to the relationship between damselfish and acroporids, coral health and mortality, and growth of the genotypes in those environments. Taking into consideration that the first step to evaluate ecological processes is the adequate and rigorous description of patterns of spatial–temporal variation of relevant variables, the objectives of this work were: (1) to assess the potential for rapid recovery of acroporids in the La Parguera Natural Reserve (LPNR) through the characterization of spatial and temporal patterns of coral linear extension, mortality, and volumetric expansion; and (2) to assess the spatial and temporal patterns of variation of algal accumulation and fish/invertebrate assemblages associated with the two species of *Acropora* considered in this study. Understanding population demographics of *A. cervicornis* and the hybrid *A. prolifera* in southwest Puerto Rico will aid future restoration and conservation efforts that could eventually support the recovery of this important structural complexity. Historically, acroporids have provided the essential complexity that: (1) allowed successful recruitment and development of juvenile reef fish and invertebrates (including commercially important species); (2) contributed to coastal protection and sediment stabilization; and (3) fostered biodiversity and ecosystem productivity. As such, these foundational species have received much attention from NOAA’s coral reef management plans, the Endangered Species Act and the IUCN Red list (Weil et al., 2002; Acropora Biological Review Team, 2005; Aronson et al., 2008). Additionally, the hybrid *A. prolifera* has been described in previous studies as having high hybrid viability, lower adult mortality, lower disease prevalence, higher tolerance to variable environmental conditions, and higher branch density than *A. cervicornis* (Bowden-Kerby, 2008; Fogarty, 2012. These investigations, along with results presented here, highlight the potential ecologically role that *A. prolifera* has in coral reef ecosystem recovery given the present state of coral reef ecosystems.

## Materials & Methods

### Site selection and field surveys

**Figure 1:**
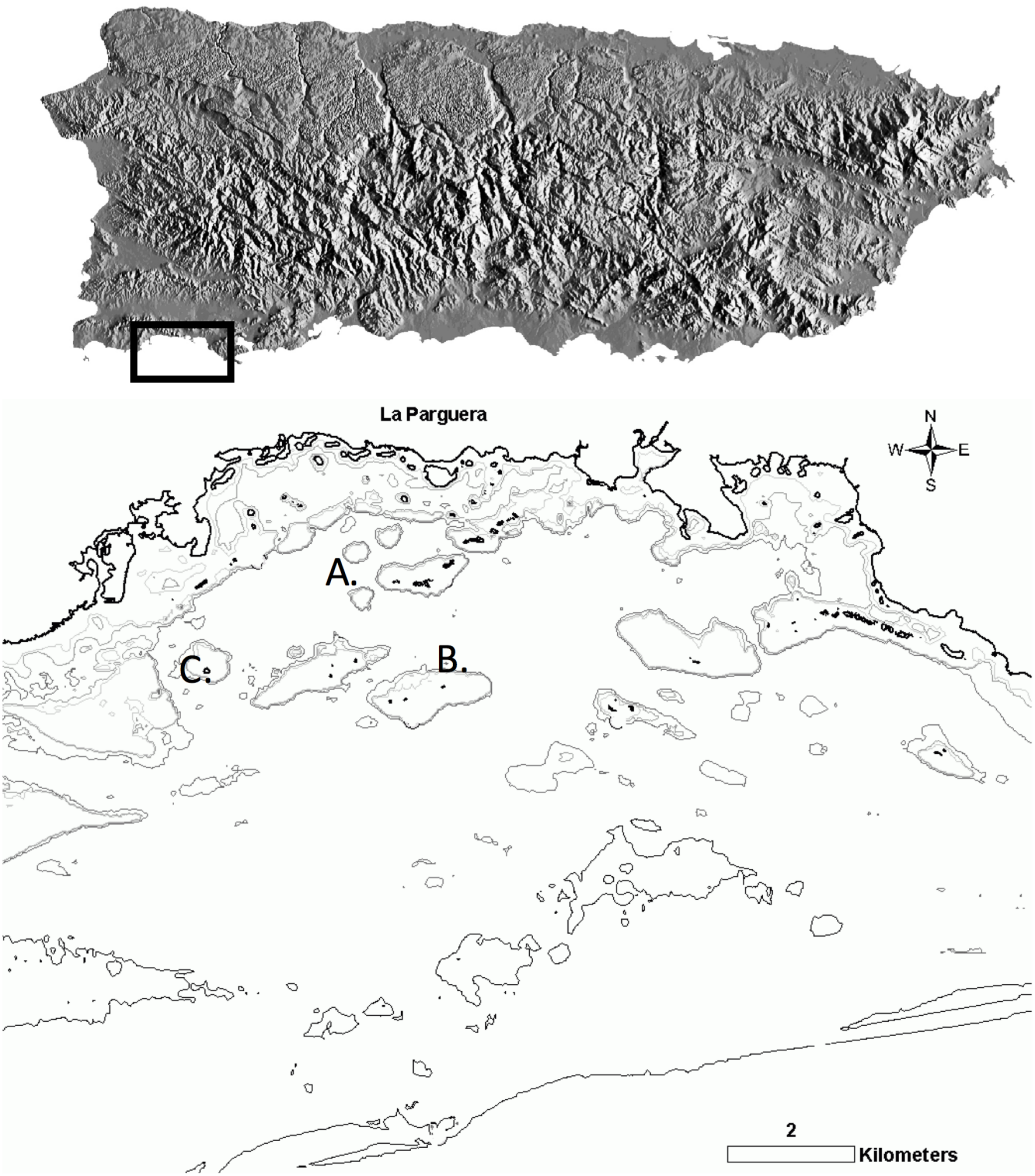
Survey sites in La Parguera Natural Reserve (LPNR) on the southwest of Puerto Rico. (A) Mario (17°95′N, −67°05′W), (B) Media Luna (17°95′N, −67°05′W), (C) San Cristobal (17°93′N, −67°11′W).

After five years of little to no bleaching events or major disease outbreaks observed, populations of *A. cervicornis*, *A. palmata* and their hybrid *A. prolifera* have re-bounded in several sites along the southwest coast of Puerto Rico (Lucas & Weil, 2015). Out of those multiple sites, three were chosen in the La Parguera Natural Reserve (PNR), Puerto Rico (Fig. 1). Criteria to define the sampling sites for our study, were based on: (1) the existence of long-term observations of environmental conditions, habitat type, and colony health status; and (2) relative abundance of *A. cervicornis* and *A. prolifera* (Lucas & Weil, 2015).

Small, medium and large thickets of *A. cervicornis* and its associated hybrid *A. prolifera* were abundant at San Cristobal (17.93N, −67.11W) and Mario reefs (17.95N, −67.05W), while only *A. cervicornis* was present at Media Luna (17.95N, −67.05W). San Cristobal and Media Luna are shallow (1–2 m) back reef habitats with a coarse rubble and sand substrate. Wave action is mitigated by the sheltering effect of a continuous, emergent linear fringing reef to the east and south. Mario is a ring shaped patch reef with a shallow breakage area to the east and southeast, and a shallow, sand and rubble lagoon (3–4 m deep) with an eastern open narrow channel (Fig. 1).

**Figure 2:**
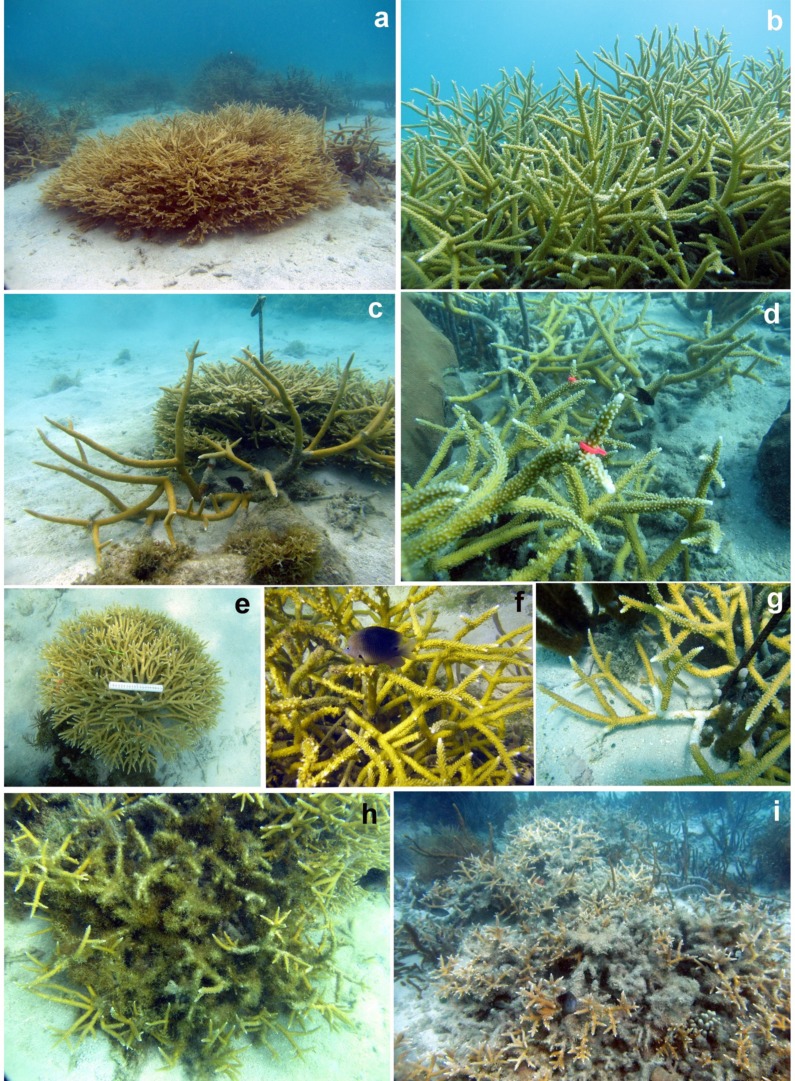
Photographs of the two taxa showing the different morphologies, tagged branches, damselfish bites, disease signs and algae smoothering. (A) Compact thicket of *A. prolifera*. (B) Well developed *A. cervicornis* colony. (C) Open, thick branches of *A. cervicornis* growing side by side with *A. prolifera*. (D) Tagged branches of *A. cervicornis*. (E) Calibration rod on *A. prolifera*. (F) Damselfish and bite marks in *A. cervicornis*. (G) White band disease at the base of branches in *A. cervicornis*. (H) Damsel fish algal lawn. (I) Algae smothering of a large colony of *A. cervicornis*. Photos courtesy of Ernesto Weil.

*A. prolifera* has a shorter and denser branching pattern compared to the open, long branches of *A. cervicornis*. It grows into compact, low profile thickets with little inter-branch space (Fig. 2A). Although intermediate morphotypes of the hybrid have been reported, all those observed and selected for these sites appeared very similar in shape and branch size/density. *A*. *cervicornis* presented two different growth morphologies (ecomorphs) in the area: one characterized by thin, long and slender, yellowish branches (Fig. 2B); and a robust, wide-spaced, orange-brownish with mostly long and thicker branches forming open, less compact thickets (Fig. 2C). Only the thin and more abundant ecomorph was used in this study. Healthy-looking colonies of similar size (1–2 m in diameter) were selected during preliminary observations one month prior to the commencement of this investigation (August 2015) and marked with a numbered tag on a rebar hammered into the adjacent sandy substrate. Each plot in San Cristobal and Mario contained both taxa in close proximity (<2 m), while Media Luna only had *A. cervicornis*, though all plots were at least five m apart to prevent overlapping of damselfish territories. Each site was visited monthly from September 2015 to August 2016. All colonies tagged in San Cristobal, Media Luna and Mario reefs seemed to be the product of new sexual recruitment after the bleaching event of 2010. Other colonies in these areas have survived partial mortalities and disease since 2005, when they grew from new sexual recruits. All colonies in these three localities were wiped out by the intense thermal anomaly of 2005, which produced widespread bleaching and disease outbreaks in the area (Weil, Croquer & Urreiztieta, 2009). Right after we finished our surveys, hurricane Mathew passed 250 km south of Puerto Rico and produced significant swells that uprooted and fragmented all colonies in Media Luna, but did very little damage (few colonies turned over) at Mario or San Cristobal.

### Growth

Volumetric growth for each colony was measured and photographed from the top and from the side with a 20-cm scale at the beginning and end of this study to obtain overall volumetric growth differentials and mortality areas. The individual branches were measured (five replicates per taxa/colony) for apical growth (cm). Each branch was randomly selected in each individual colony and marked at the base with a thin, colored cable tie (Fig. 2D). The length of the branch was measured with a plastic ruler (cm) and photographed (Fig. 2E). This was time zero for the assessment of linear growth and volumetric increase. Linear growth rates and total volume of each colony was determined using Coral Point Count (Kohler & Gill, 2006) with excel extensions as outlined by Kiel, Huntington & Miller (2012).

### Algal cover

Three branches were marked for algal growth with fluorescent string in each colony of *A. cervicornis* to assess algal accumulation and associated coral mortality over time (Figs. 2H and 2I). Branches were chosen based on the presence of already established algal accumulation (>1 cm). *A. prolifera* was not marked because prior to this study and, also throughout, there was no conspicuous algal accumulation on *A. prolifera* branches, and marking the branches may have artificially allowed algae colonization near healthy tissue. Many *A. cervicornis* colonies that were surveyed prior to the investigation did not show signs of heavy algal accumulation; therefore only three branches were marked, which was the most branches already with algal overgrowth that could be found at each plot during the initial survey. Algal overgrowth was measured for *A. cervicornis* only (cm) (3 replicates per plot, 10 replicates per site), and algal composition (i.e., crustose coralline algae or *Dictyota* sp.) was identified.

### Associated fish and invertebrate assemblages

The composition and abundance of fish and invertebrate assemblages (herbivores, omnivores and coralivores) associated with each plot was evaluated monthly. Each plot (10 replicates per site) was treated as a whole rather than each colony separate because of the behavioral nature of roving herbivores, which may shortly visit each taxa in passing. Additionally the highly branching nature of *A. cervicornis* creates a more accessible refuge for several fish and invertebrate species as opposed to *A. prolifera,* however the proximity of each colony per plot favored potential mobility between the two. Once each plot was located and before measurements were taken, a timed underwater visual census commenced when the diver was approximately 2–3 m from the colonies. The diver then recorded roving herbivores, damselfish and omnivores present during a five minute census. After the fish were counted, the invertebrates present were also located and counted. All surveys and observations were done using SCUBA.

### Tissue mortality

Algae do not typically settle on healthy tissue, and several health-impairing factors contributing to tissue mortality help to pave the way for algal overgrowth and smothering. As such, each taxa per plot (2 colonies per plot, except Media Luna, 10 replicates per site) was also examined each month for signs of, bite marks (predation from coralivores or herbivore grazing) and disease (white band and/or red band). These were recorded as present or absent for the entire colony, assuming the whole colony is equally susceptible to each of these variables. Overall mortality was then estimated for all replicates as a percent of the entire colony exhibiting signs of algal overgrowth on already dead tissue or branches that have already succumb to disease.

### Statistical analysis

Multifactorial, mixed analyses of variance based on permutations were done to test various hypotheses related to potential differences between species and whether those differences were consistent across different spatial and temporal scales. The sources of variation in this experiment were:

Season = fixed factor with four levels (spring, summer, fall and winter). This factor was included in the model to evaluate whether the response variables considered in this study responded to clear changes of natural environmental conditions.

Sampling time = random factor nested within Season with 2 to 4 levels depending of season. This factor was included in the model to avoid simple pseudo replication (see Hurlbert, 1984) when comparing seasons.

Taxa (species) = fixed factor with two levels (*A. cervicornis* and *A. prolifera*). This factor is orthogonal to all above and was considered in the model to test the main null hypothesis of this study (i.e., no differences between species).

Site = random factor with three levels (Mario, Media Luna and San Cristobal). This factor is orthogonal to all above and was considered to test the generality of potential differences observed between species.

Response variables analysed with the above model consisted of univariate and multivariate data sets. Univariate response variables were: linear growth and algal accumulation, whereas the multivariate data set was the structure and composition of fish assemblages. For univariate analysis, the complete model described above was considered, and the sums of squares (Type 3) for each source of variation were estimated by calculating Euclidean distances among all pairs of individual observations (i.e., replicated branches per colony, time, season, site and species). In the case of multivariate analyses, the factor species was not considered due the proximity of adjacent experimental units (i.e., colonies) and the mobility of the response matrix (i.e., fish assemblages). In this case, a Bray-Curtis similarities matrix calculated on the square rooted transformed data was the basis of multivariate analyses. Data was square root transformed to down weight the contribution of abundant species. Null distributions for univariate and multivariate analyses were constructed using 999 permutations of the residuals under a reduced model. All analyses (univariate and multivariate) were performed using the routine PERMANOVA (Anderson, 2017). Patterns of multivariate variation across sites and times were illustrated using Principal Coordinate Analyses (PCO) using the routine PCO. All routines were done with the software PRIMER V7 and PERMANOVA add on Clarke & Gorley (2015).

## Results

### Growth

**Figure 3:**
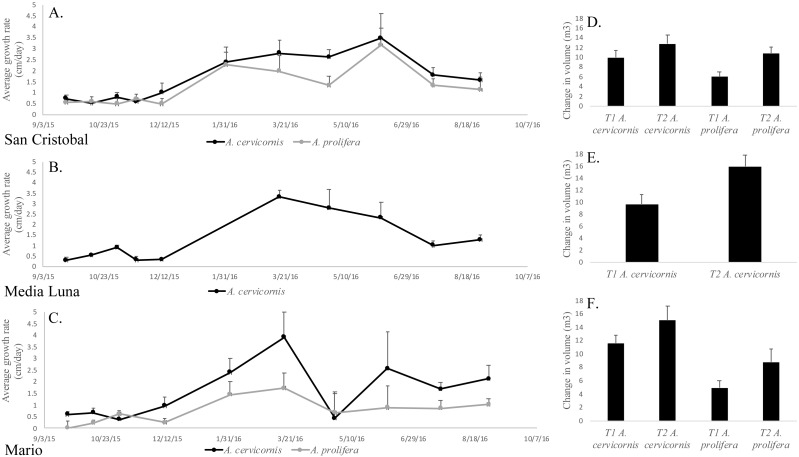
Mean linear growth and volumetric increase in the two taxa in the different localities. (A) Average linear growth rate (cm/month + SE) of *A. cervicornis* and *A. prolifera* colonies in San Cristobal. (B) *A. cervicornis* in Media Luna. (C) *A. cervicornis* and *A. prolifera* in Mario. (D) Average volumetric grow (m^3^/year + SE) for *A. cervicornis* and *A. prolifera* colonies in San Cristobal. (F) *A. cervicornis* in Media Luna (E), and *A. cervicornis* and *A. prolifera* in Mario.

Permutation analysis of variances (PERMANOVA) showed a statistically significant first order interaction between ‘site’ and ‘season’ for growth rates (Table 1), indicating that temporal differences depended on the site being sampled. Nevertheless, a posteriori comparisons of this interaction showed a significantly higher growth rate during winter/spring (for both species and in all sites) compared to summer/fall (Fig. 3, Table 1). The only exception (April 2016) at Mario, showed a significant decrease, but this was due to the breakage of tips of tagged branches (Fig. 3) in a few colonies. In addition to the first order interaction described above, there was a significant and independent effect of species, where linear growth rates were consistently and significantly higher in *A. cervicornis* [3.1 ± 0.44 cm/month (= 37.2 cm/y)] compared to *A. prolifera* [2.6 ±0.41 cm/month (= 31.2 cm/y)] (Table 1, Fig. 3). Unexpectedly, linear growth was significantly higher during Winter-Spring compared to Summer-Fall for both taxa (3.5 ± 0.58 vs. 0.53 ± 0.15 cm/month in *A. cervicornis,* and 2.43 ± 0.71 vs. 0.27 ± 0.20 cm/month in *A. prolifera*, respectively). This seems to be a clear indication of the diversion of resources from growth into reproduction (production of eggs) and spawning in both taxa. Growth rates were lower for both species at the deeper and more biologically compromised (algae overgrowth, damselfish, predation and disease (Figs. 2F–2I)) habitat in Mario, compared to shallow San Cristobal and Media Luna (Fig. 3). Nevertheless, in all sites and for both taxa, temporal variation in growth rates resulted in absolute increases of the total volume of the colonies at the end of the study (Figs. 3D–3F). On average, colonies of *A. cervicornis* increased by 4.17 ± 1.71 m^3^/year across all three sites and *A. prolifera* increased by 4.39 ± 1.34 m^3^/year across two sites.

**Table 1:** PERMANOVA results based on Euclidean distances on mean growth rate for the two acroporids, to test for the effect of: Season (Se), Fall, Winter, Spring and Summer; Sampling time (Ti), 2 to three nested per season; Sites (Si), Mario and San Cristobal (no A. prolifera found in Media Luna) and Species (Sp), *A. cervicornis* and *A. prolifera*.

**Source of Variation**	**df**	**MS**	**Pseudo F**	***p*(perm)**
Season = Se	3	0.008	1.09	0.454
**Site = Si**	**1**	**0.014**	**19.32**	**0.006**
**Species = Sp**	**1**	**0.017**	**10.99**	**0.006**
Time(Se) = Ti(Se)	7	0.002	3.36	0.077
**Se × Si**	**3**	**0.006**	**8.09**	**0.016**
Se × Sp	3	0.001	0.89	0.570
Si × Sp	1	0.001	0.74	0.419
Ti(Se) × Si	6	0.001	0.40	0.883
Ti(Se) × Sp	7	0.001	0.88	0.576
Se × Si × Sp	3	0.001	0.99	0.458
Ti(Se) × Si × Sp	6	0.001	0.60	0.738
Res	317	0.002		
Total	358			

### Algal cover

**Figure 4:**
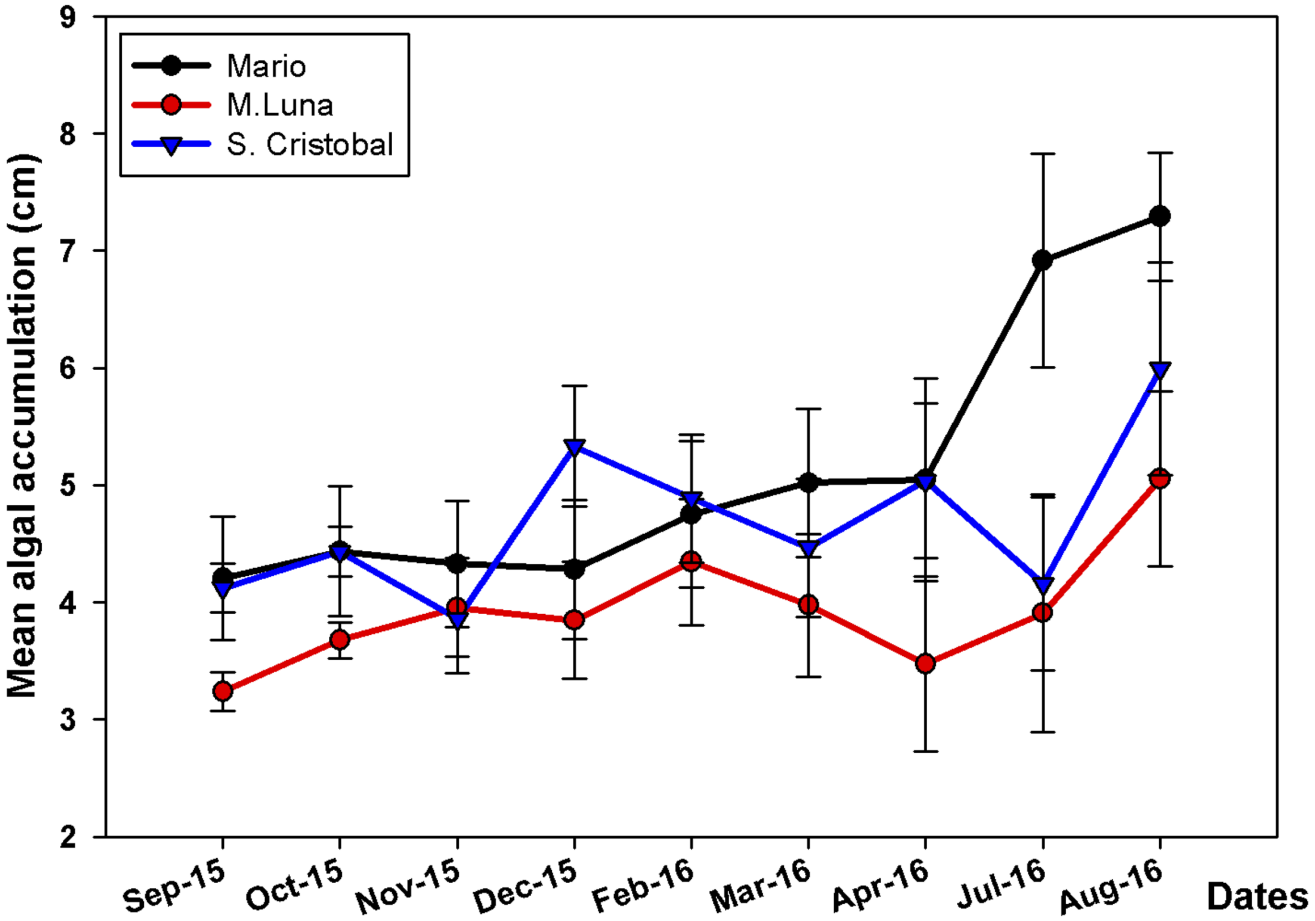
Mean algae accumulation on *A. cervicornis*. Mean algae accumulation (cm + SE) on *A. cervicornis* in the three localities surveyed. There is high algae productivity during the warmer months compared to colder winter–spring months. Only Mario showed a significant increase in July–August 2016.

*A. prolifera* did not have any conspicuous macro-algae overgrowth during the time of the study; observations in other areas indicate that algae-overgrowth is not common on colonies of *A. prolifera* in La Parguera. In contrast, algal-smothering is a major problem in *A. cervicornis*. PERMANOVA results showed that there was a significant and independent (i.e., no interaction) effect of the main factors (Site and Season) on the accumulation of algae (Table 2). Consistent patterns of algae cover expansion were observed across seasons, with zero or low expansion from December until May, the cold-water months, and then, sharp increases during the summer (Fig. 4). Higher algae cover expansion during the summer months was observed in colonies at Mario reef, where higher densities of the sea urchin *Echinometra lucunter*, higher proportion of fish bites, and higher prevalence of disease were observed compared to the other two sites. Conspicuous changes in algal species composition occurred when *Dictyota* spp. overgrew branches during the summer months (Figs. 2H and 2I).

**Table 2:** PERMANOVA results based on Euclidean distances on algal extension (cm/month) for the two acroporids, to test for the effect of: Season (Se), Fall, Winter, Spring and Summer; Sampling time (Ti), two to four nested per season; Sites (Si), Mario, San Cristobal and Media Luna. For these analyses, algal growth was not discriminated among species.

**Source of variation**	**df**	**MS**	**Pseudo F**	***p*(perm)**
**Season = Se**	**3**	**26.816**	**6.62**	**0.002**
**Site = Si**	**2**	**10.021**	**4.93**	**0.037**
Time(Se) = Ti(Se)	7	1.5561	0.78	0.622
Se × Si	5	3.2125	1.57	0.239
Ti(Se) × Si	11	1.9667	0.65	0.799
Res	227	3.0155		
Total	255			

### Associated fish and invertebrate assemblages

A total of twelve herbivorous fish species were identified and recorded based on their regularity of encounters at all sites (Table 3). Roving herbivore observations were only recorded at the level of plot due to their fleeting grazing patterns. The damselfish, however, was also recorded at plot level due to its high site fidelity, the low number of observations of both macro-algae and damselfish for plots containing *A. prolifera*, and the proximity of both acroporids at each plot. Six of the herbivores noted were damselfish from the genus *Stegastes*, except for the yellowtail damsel *Microspathodon chrysuris*. All damselfish were observed in the colonies at each site at least once, however, the highest frequency of occurrence was by the three-spot damselfish, *Stegastes planifroms*, which had algal lawns in at least fifty percent of the *A. cervicornis* colonies at each site in all surveys. The highest relative proportion of *S. planifrons* (35.09%, Table 3) was found in San Cristobal.

**Table 3:** Relative species abundance of associated organisms observed in the two *Acropora taxa*.

Species	Mario	San Cristobal	Media Luna
*Stegastes planifrons*	18.19	35.09	18.50
*Stegastes diencaeus*	1.64	12.72	3.85
*Stegastes leucostictus*	0.94	0.39	1.12
*Stegastes partitus*	1.64	3.08	0.87
*Stegastes variabilis*	1.29	0.26	2.48
*Microspathodonchrysurus*	0.70	0.64	1.99
*Scarus taeniopterus*	7.86	8.74	11.42
*Scarus iseri*	3.40	6.30	5.96
*Sparisoma viridi*	1.64	4.50	4.14
*Sparisoma aurofrenatum*	0.12	0.39	0.37
*Sparisoma chrysopterum*	1.06	0.00	0.12
*Acanthurus coeruleus*	3.29	2.70	5.09
*Haemulon flavolineatum*	4.11	12.72	3.35
*Holocentrus adscensionis*	0.35	2.70	4.35
*Hypoplectrus chlorurus*	2.00	0.64	0.75
*Hypoplectrus puella*	0.35	0.26	0.12
*Thalassoma bifasciatum*	0.94	0.64	5.71
*Halichoeres bivittus*	3.29	5.91	6.46
*Cephalopholis fulva*	0.24	0.00	0.00
*Echnometra lucunter*	46.83	1.93	22.97
*Eucidaris tribuloides*	0.12	0.39	0.37

Roving herbivores that entered the plots solely for grazing comprised the remaining three fish taxa in this group (*Scarus* spp., *Sparisoma* spp. and *Acanthurus coeruleus*). All were observed at each site at least once, apart from *Sparisoma chrysopterum*, the redtail parrotfish. This species was not found at any time at San Cristobal, and only found infrequently at Mario and Media Luna (Table 3). Although not considered in the overall spatial and temporal analysis due to their lack of site fidelity central to this study, it is important to note that many of the parrotfish species were encountered regularly at all sites. In fact, except for *S. planifroms, Scarus taeniopterus* was observed at higher relative proportions (7.86% Mario, 8.74% San Cristobal and 11.42% Media Luna) than most pomocentrids at all sites, (Table 3). Six species of omnivorous fish were also found regularly in association with *Acropora* colonies at all sites (Table 3); two of which, *Haemulon flavolineatum* and *Holocentrus adscensionis*, were most frequently observed sheltering within the branches. *Cephalopholis fulva* was also considered within this category given that only the juvenile life stage was encountered, albeit not frequently. This fish was also found sheltered beneath the branches at the base of colonies only at the Mario site.

**Figure 5:**
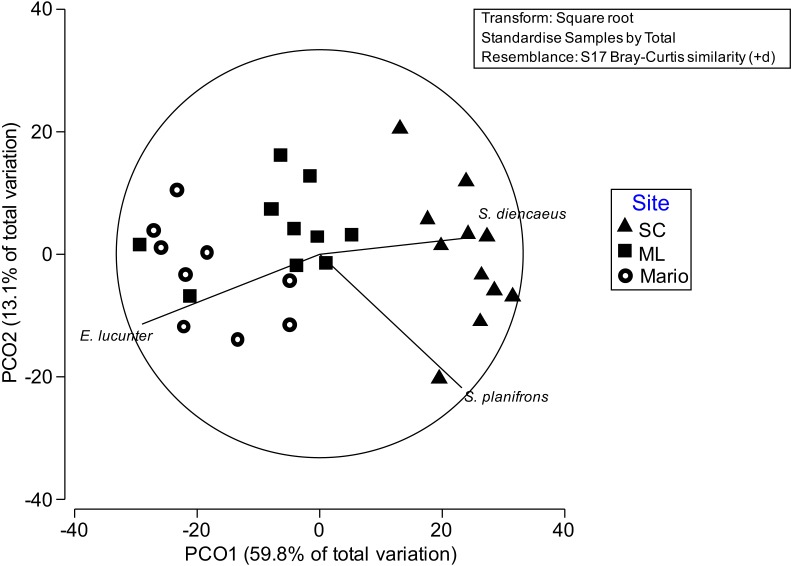
Principal Coordinate ordination plots by sampling time. Principal Coordinate ordination plots by sampling time (PCO1) and site (PCO2). Vectors indicate species that were correlated (>0.7) with either site or time of ordinations. Mario (open circles) reef had higher densities of *E. Lucunter* and the two damselfish *S. planifroms* and *S. diencaeus* were more abundant in San Cristobal (triangles). Media Luna (squares) had low presence of these taxa.

Analyses on patterns of spatial and temporal variation of these assemblages showed a significant effect due to site, but not to the temporal factors (Table 4). These differences were mainly due to damselfishes (especially *S. planifrons* and *S. diancaeus*), which were more abundant in San Cristobal than in Mario, whereas the sea urchin *E. viride* was more abundant in Mario than San Cristobal (Fig. 5). Media Luna had intermediate abundances of these three species. Contrary to what was observed for growth and some health indicators, there were no clear temporal patterns of variation of fish assemblages associated with acroporids in the study sites (Fig. S1). Not only were different temporal trends observed in all sites, but also species correlated with those patterns were not the same. An interesting exception to this observation is the French Grunt (*Haemulon flavolineatum)* that appeared in all sites around the second and third sampling time (Fig. S1). Damselfish densities at all sites remained relatively consistent throughout the year regardless of changes in algal composition.

**Table 4:** PERMANOVA results based on Bray Curtis dissimilarities (Squared root transformed and standardized by total) on assemblages of fish associated with the acroporids and testing for the effect of: Season (Se), Fall, Winter, Spring and Summer; Sampling time (Ti), two to four nested within season, and Sites (Si), Mario, San Cristobal and Media Luna.

**Source of Variation**	**df**	**MS**	**Pseudo F**	***p*(perm)**
Season = Se	3	5415.40	1.57	0.068
**Site = Si**	**2**	**41617.00**	**21.30**	**0.001**
Time(Se) = Ti(Se)	8	2384.90	1.22	0.216
Se × Si	6	2771.10	1.42	0.107
Ti(Se) × Si	13	1955.30	1.02	0.422
Res	252	1909.30		
Total	284			

The red sea urchin, *Echinometra viride*, was the only macro-invertebrate living within *A. cervicornis* colonies across all sites and times (Table 3, Fig. 5). *Eucidaris tribuloides* was observed occasionally. *E. viride* had higher relative abundances at Mario and Media Luna compared to San Cristobal (Table 3). The number of urchins counted per colony at San Cristobal remained low for all plots and all sampling times throughout the year, and no more than four were counted within a colony at any time. This is a sharp contrast to Media Luna and Mario where up to ten urchins per plot and sampling time were quite common, and counts into the low thirties at Mario were recorded on two occasions and two separate plots.

### Tissue mortality

**Figure 6:**
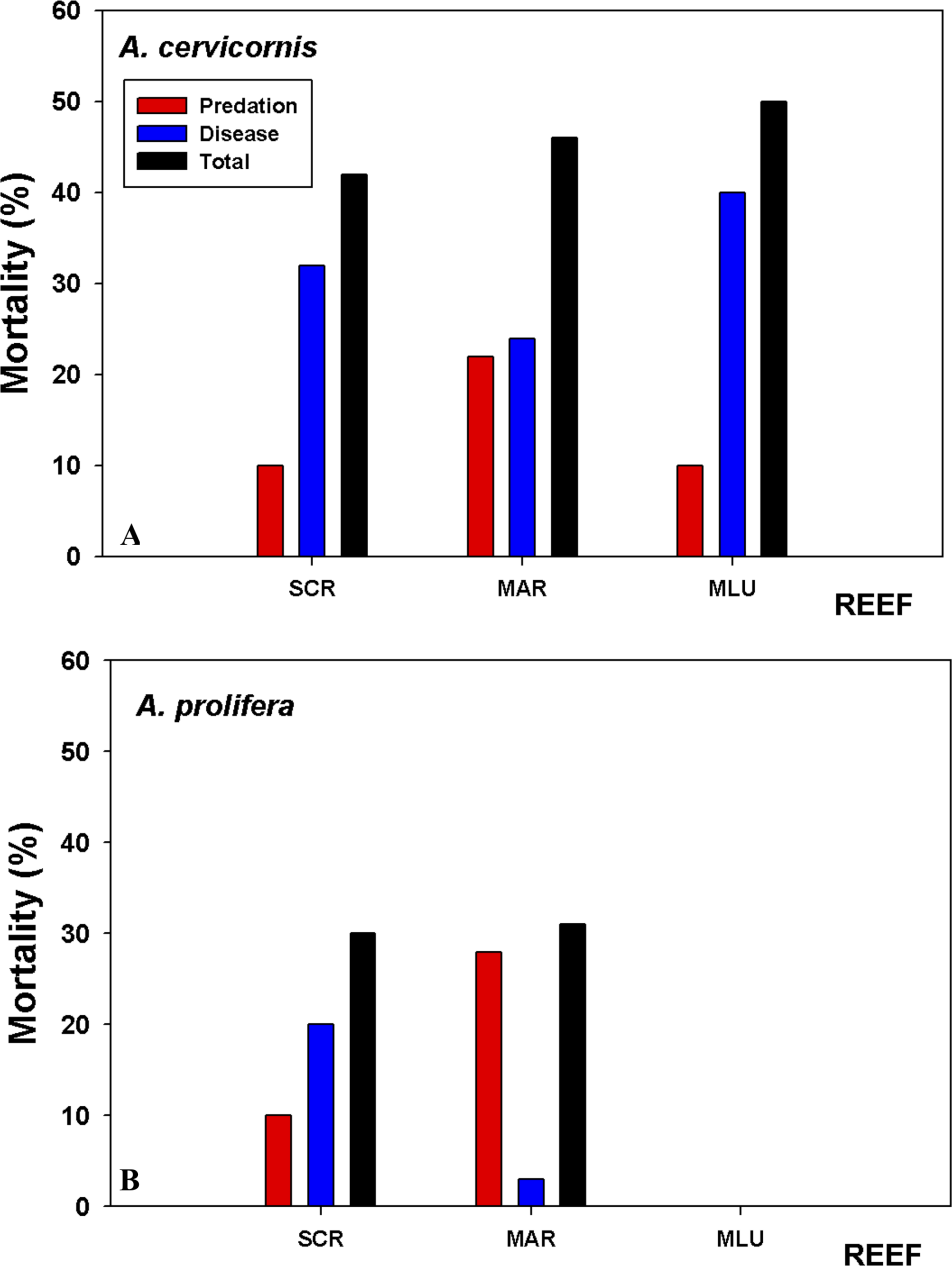
Coral tissue mortality by predation and disease. Coral tissue mortality (predation + disease) based on relative cover (%) of dead versus live tissue in the tagged branches averaged across colonies estimated from the difference in tissue mortality at the beginning (09/2015) and end of the study (08/2016). (A) *A. cervicornis* suffered the highest overall mortality at Media Luna (MLU) and San Cristobal (SCR) over the one-year sampling period. (B) *A. prolifera* experienced higher predation at Mario but similar total mortality at both sites. Disease impacted *A. cervicornis* more in all three reefs.

Tissue mortality (estimated as the relative cover of dead tissue compared to live tissue), varied between the species and across sites (Fig. 6, Table S1). The significant sources of variation for mortality was period and site (i.e., start to end of the study, and site (Table S1)). Percentage cover of dead areas increased in all sites and for both species through the length of the study, with the greatest increase in *A. cervicornis* at San Cristobal (Fig. 6). *A. prolifera*, had lower overall mortality rates, yet the increase in mortality over the study period was higher compared to *A. cervicornis* for Mario colonies, 31% versus 22% increase, respectively. When algal composition changed to high abundances of the foliose *Dictyota* spp. in *A. cervicornis*, a marked increase in white band disease (WBD) and coral tissue mortality was observed. White band disease (WBD) was common among all sites. Another condition termed “*Acropora* red band” which has been observed for some time now in LPNR (E Weil, pers. obs., 2016) was common in Media Luna and San Cristobal colonies, largely affecting *A. cervicornis*. However, no tissue mortality associated with this condition was observed. In general, white band disease was present in both taxa in all sites but, it was significantly less prevalent on *A. prolifera* in Mario (Fig. 6).

*A. cervicornis* showed significantly higher (*P* < 0.022) proportion of colonies with predation signs compared to *A. prolifera* in all sites, with between 90% and 100% of colonies having evidence of predation. Only 30–40% of *A. prolifera* showed evidence of predation during the initial survey time, with the highest increase to 71% only at Mario (Table S1). All surveyed colonies of *A. cervicornis* showed similar signs of predation throughout the experiment (damselfish bites, snail and fireworm). Overall, total proportion of tissue mortality was considerably lower in *A. prolifera* compared to *A. cervicornis* (Fig. 6) because of lower disease prevalence, lower predation and no macro-algal overgrowth.

## Discussion

As worldwide coral reefs continue to degrade, measures to reverse this trend and reduce ecological and socio-economic phase shifts has become paramount (Pandolfi et al., 2003; Hughes et al., 2018). This is especially true within the Caribbean after the disappearance of the acroporids and *Diadema* due to the onslaught of disease epizootics, bleaching events, overfishing and other anthropogenic impacts (Aronson & Precht, 2001; Precht et al., 2002; Weil & Rogers, 2011; Jackson et al., 2014; Weil, Rogers & Croquer, 2017). Acroporids are the fastest growing coral genus in both the Caribbean and the Indo-Pacific, providing the physical and biological foundation for many shallow water coral reef communities (Lirman, 1999; Aronson & Precht, 2001). Overall, significant recovery of acroporid populations has not been observed for at least 35 years. Only *A. palmata* have been reported and/or observed to have had good recovery in just a few localities (Lucas & Weil, 2015). Even today, acroporid populations in most localities have not recovered to pre-1980’s levels. Threats to both species have remained high, and in some cases increased (e.g., thermal anomalies, recurrent disease, bleaching events, storms, predation, algal overgrowth, lack of sexual reproduction) with continuous local anthropogenic stress in coastal areas (Weil et al., 2002; Aronson et al., 2008; Weil & Rogers, 2011; Aronson & Precht, 2016). Since populations are susceptible to many different stressors, any one severe disturbance event (or synergistic events) could lead to local and/or geographic extinction, therefore, it has been suggested that these species would not recover easily from the depauperated, locally limited, extant populations (Aronson et al., 2008; Goergen et al., 2019).

Results of this study show the seasonal growth dynamics of both *A. cervicornis* and *A. prolifera* and the impact of natural, common stressors over a short period of time (one year). Both taxa showed high growth rates averaging 37.2 cm/y for *A. cervicornis* and 31.2 cm/y for *A. prolifera*, which translates into colony-volumetric increases of up to 4 m^3^ per year (Fig. 3). These rates are significantly higher than those reported for *A. cervicornis* in Barbados (14 cm/y) (Lewis, 1974) and in Jamaica (12–26 cm/y) (Tunnicliffe, 1983) and explain in part the fast growth and colony sizes observed from 2010 to the present study. However, these rates are similar and/or significantly lower to growth rates reported for different genotypes of asexually produced, nursery and field fragments in Florida, the Dominican Republic and other localities (25.6–80.6 cm/y) (Lirman et al., 2014; Schopmeyer et al., 2017).

In contrast to our results, Bowden-Kerby (2008) reported higher growth rates for *A. prolifera* compared to *A. cervicornis* for the same area in La Parguera, which indicates that growth rates are probably highly variable across habitats and over time for both taxa, and single-year data might be limited to address growth and survivorship over mid- to long periods of time. Good environmental conditions since 2010 might explain the significantly higher growth rates of these two taxa for this period compared to previous data, and to other areas in the Caribbean. Changes in light quantity and quality and water motions affect growth rates and growth morphologies in most coral species. The San Cristobal thickets are growing in shallow (0.5–2 m), well-illuminated, coarse sandy habitat with good water circulation and is 1.9 km from the closest mangrove coastline. In comparison, in Mario reef, which is about 2.2 km from the mainland, populations are growing in deeper (4–5 m), coarse sandy bottom and hence, the lower growth rates observed.

Branches grew significantly faster during the cold season (winter and spring) compared to summer and fall in both species. This is consistent with results reported for *A. cervicornis* (Gladfelter, 1984; Childcoat, 2004) and *Pocillopora damicornis* (Ward, 1995), which might be related to the increase in energy and resource allocation into sexual reproduction, which ends with massive spawning events in late August and September (Vargas-Ángel et al., 2006; Van Woesik, Lacharmoise & Koksal, 2006; E Weil, pers. obs., 2016). Furthermore, the negative interaction between algal overgrowth and linear extension during the summer months is reversed during winter and spring when algal growth is slower and WBD usually arrests (Gil-Agudelo, Smith & Weil, 2006), thus favoring faster linear extension rates of branches with much lower tissue mortality. Winter and spring months are relatively rainless (dry season) in southwest Puerto Rico, therefore nutrient input from rain water runoff is minimum, thereby limiting algae growth. High nutrient concentrations (nitrate and phosphate) for example, reduce growth rates in *A. cervicornis* (Renegar & Riegl, 2005) and increase potential algal overgrowth and tissue mortalities.

In addition, *A. prolifera* has a tighter branch structure that seems to be not suitable for damselfish algal lawns (Fig. 2), and significantly reduces tissue loss and open wounds (susceptible to infections/diseases) compared to *A. cervicornis*. This taxon also showed no algal overgrowth, and significantly less predation signs and tissue mortality compared with *A. cervicornis*, which had significantly higher tissue losses due to damselfish territories, higher disease prevalence, higher predation and overgrowth by macroalgae. Lower disease prevalence in *A. prolifera* might indicate it is more resistant to diseases. Seasonal variation in all these variables needs further temporal replication, since storms, diseases, thermal anomalies, bleaching, predation and other anthropogenic impacts could skew results from a single season or year of study. Furthermore, results are similar to those just published for a multi-year study in Florida (Goergen et al., 2019). These authors also pointed out that substantial length of time between major disturbances seems to be needed for populations of *A. cervicornis* to recover, especially when the frequency and intensity of major stressors (bleaching, disease, storms, algae overgrowth, predation, etc.) linked to global warming (GW) and anthropogenic activities are increasing. It is important to note that while *A. cervicornis* has a higher growth rate, their elongated and branching structure makes them more susceptible to breakage during localized storm events. On the other hand, *A. prolifera* could potentially persist through mild to moderate storm events due to its skeletal density and observed hardiness and continue to provide essential fish habitat and further reef recovery.

Despite having a higher rate of disease prevalence, mortality and predation signs compared to the other two sites, San Cristobal populations consistently showed higher growth rates, which translated into higher growth and overall survivorship of both taxa. This fact potentially makes it an optimum site for future nursery or propagation activities of the acroporid species complex in the LNPR. In contrast, *A. cervicornis* was also growing fast in Mario, but it was barely keeping ahead of the mortality produced by disease, predation and algal factors, thus any increase, or additional stress that affects growth, (e.g., bleaching, an increase in tissue mortality or a combination of these) would likely tilt the balance to total branch and colony mortality. This has been the case in Mario over the last 10 years, where we have observed recovery of colonies for some time, and then, a total crash of the *A. cervicornis* population after a hot summer with mild bleaching, slight increase in WBD prevalence, and/or increased densities of damselfish, fireworm and sea urchin predation.

Although the damselfish farming behavior encourages the continued growth of algae, their preference for turf algae may slow the growth of later stages and more foliose species. *Dictyota* species were present, but, in most cases, in much lower abundance than the farmed filamentous algae. Later stages of foliose algae have a greater potential to negatively affect the coral colony itself and are not as palatable to most herbivores (Littler, Taylor & Littler, 1983). Algal succession by more aggressive and dominant species may be subdued by the farming behavior of damselfish (Ferreira et al., 1998), and thus lead to a continual presence of early stages of filamentous algae, rather than the more foliose species that could potentially smother the recruiting and recovering acroporids. This deterrence, as well as the potential grazing habitat, may provide enough preferential resources and habitat to maintain the reef environment in an intermediate healthy steady state, and continue to support the diversity of acroporid-associated fish and invertebrates. Although the prevalence of disease and possible bleaching may also have a negative future impact, the observed fish and invertebrates within the reef patches examined may provide the reef system with the balance it requires to overcome potential phase shifts in this localized area. Although *Diadema* were not encountered in our sample plots, the other species of urchins observed may play an important role in deterring the occurrence of algal phase shifts. The tissue loss occurring without the subsequent algal accumulation could, on a short temporal basis, be attributed to urchin grazing, but in the long term could signify a more positive outcome for the recovery of healthy tissue given that urchins are not deterred by damselfish nor are they as particular about the algae they feed upon (Littler, Taylor & Littler, 1983). There is a current ongoing effort in La Parguera to restore *Diadema* to the reefs and, in turn, this algal consumption by a healthy population of reef grazers could maintain a balance between fish, algae and corals and aid in reef resilience.

An important piece of information missing in this study is the genetic diversity of these populations and how this affects growth and mortality rates (disease susceptibility). Live acroporid colonies suffered almost 100% mortality after the high thermal anomalies induced bleaching and disease outbreaks in 2005 and 2010. They have been presumably recolonized by sexual recruitment and therefore, different genomes. In this study we selected widely separated colonies (>10 m) and survey sites (>1,000 m) to avoid as much as possible the selection of clones. Being produced after the disease and bleaching impacts, we assume that most of these genotypes were less susceptible than those that died in 2005–10. This genetic inference and the frontal reef-crest sheltering properties of selected reefs are important considerations for future selection of potential nursery or re-population areas in the LPNR or elsewhere. Overall, results showed that recovery of the acroporids seems to be species and site specific, and attempts to thoroughly conserve and or restore populations of these foundational taxa need to consider the recent history of targeted populations, their genetic variability, variable environmental and ecological stressors and anthropogenic impacts before developing nurseries and/or providing further protection to natural populations of these acroporids.

## Conclusions

• *A. cervicornis* consistently grew faster than *A. prolifera* across all three study sites during our study period. Growth rates were on average lower than those reported for nursery fragments, but higher than most of those reported for wild populations in the region. Growth was higher during the cold months of the year, probably resulting from the diversion of resources for reproduction during the summer months.
• *A. cervicornis* had consistent problems with algal overgrowth, disease and higher damselfish bites and predation pressure, which resulted in higher mean tissue mortality compared to *A. prolifera*.
• San Cristobal had the healthiest and faster growing populations of both taxa. It had overall, better environmental conditions (water quality/clarity, circulation, protection from storm surge and waves, and high illumination) compared to the other two sites.
• Despite the territorial guarding behavior of the damselfish within their algal gardens, the *Acropora* patches support a diverse fish assemblage and provide shelter for several juvenile species of omnivores. Herbivory of roving herbivores and territorial damselfish acts as an intermediate disturbance which may in turn enhance the diversity of algal species, limit the acceleration of later stages of algal growth, and deter phase shifts to foliose algae dominated systems.
• Recovery of the acroporid complex seems to be species and site specific, and is highly influenced by environmental variability, so attempts to thoroughly conserve these foundational species should consider targeted populations, localities, bathymetry, ecological and anthropogenic stressors, and the genetic variability of the target populations before developing recovery/protection plans.

##  Supplemental Information

10.7717/peerj.8435/supp-1 Figure S1 Principal coordinate ordination plots (PCO) showing sampling time in three different locations Vectors indicate species that were correlated (> 0.7) with either of the two first axis of ordinations. Continued arrows indicate temporal changes.

10.7717/peerj.8435/supp-2 Data S1 Raw data set for the acropolis project in La Parguera

10.7717/peerj.8435/supp-3 Table S1 PERMANOVA results based on Euclidean distances on mortality (% of dead tissue per colony) for the acroporids PERMANOVA results based on Euclidean distances on mortality (% of dead tissue per colony) for acroporids testing for the effect of: Period (Pe) = Start and end of study; Sites (Si) = Mario and San Cristobal (no *A.* prolifera found in Media Luna) and Species (Sp) = *A. cervicornis* and *A. prolifera*.

## Additional Information and Declarations

### Data Availability

The following information was supplied regarding data availability: The raw data is in the Supplemental Files.
