# Supplementary material for: Growth dynamics in Acropora cervicornis and A. prolifera in southwest Puerto Rico
Source: PeerJ. 2020 Feb 11;8:e8435. doi: 10.7717/peerj.8435 (PMC7020822; doi:10.7717/peerj.8435)
Supplement: Figure S1 — Vectors indicate species that were correlated (> 0.7) with either of the two first axis of ordinations. Continued arrows indicate temporal changes. [file peerj-08-8435-s001.pdf]

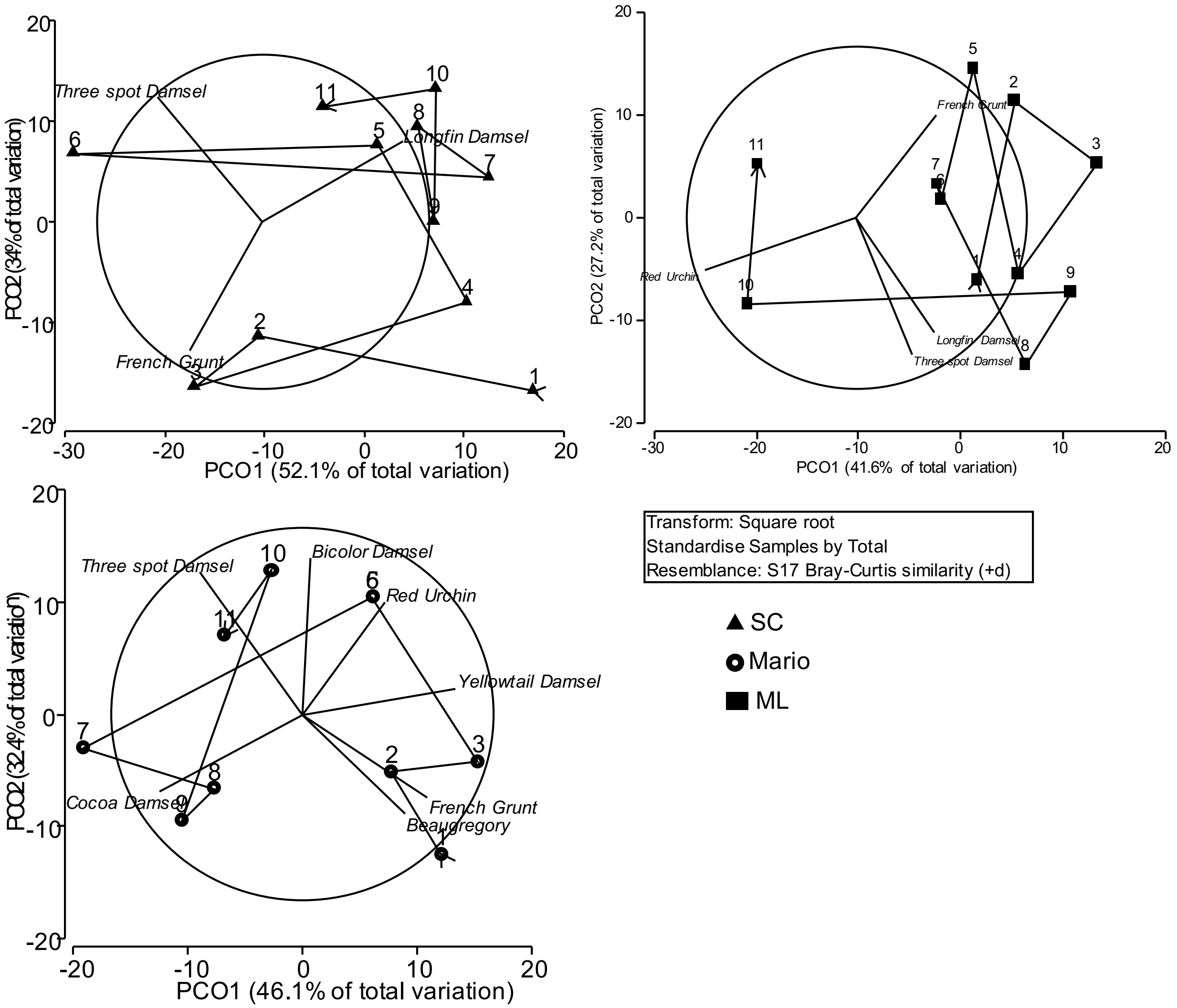

Suppl. Fig. 1. Principal coordinate ordination plots (PCO) showing sampling time in three different locations. Vectors indicate species that were correlated (> 0.7) with either of the two first axis of ordinations. Continued arrows indicate temporal changes.
