# Supplementary material for: Growth dynamics in Acropora cervicornis and A. prolifera in southwest Puerto Rico
Source: PeerJ. 2020 Feb 11;8:e8435. doi: 10.7717/peerj.8435 (PMC7020822; doi:10.7717/peerj.8435)
Supplement: Table S1 — PERMANOVA results based on Euclidean distances on mortality (% of dead tissue per colony) for acroporids testing for the effect of: Period (Pe) = Start and end of study; Sites (Si) = Mario and San Cristobal (no A. prolifera found in Media Luna) and Species (Sp) = A. cervicornis and A. prolifera. [file peerj-08-8435-s003.docx]

**Suppl. Table 1.** PERMANOVA results based on euclidean distances on mortality (% of dead tissue per colony) of acroporids, to test for the effect of: Period (Pe) = start and end of study; Sites (Si) = Mario and San Cristobal (no *A.* prolifera found in Media Luna) and Species (Sp) = *A. cervicornis* and *A. prolifera*.

| **Source of Variation** | **df** | **MS** | **Pseudo F** | ***p* (perm)** |
| --- | --- | --- | --- | --- |
| **Period = Pe** | **1** | **9539.50** | **127.26** | **0.045** |
| **Site = Si** | **1** | **1288.60** | **4.31** | **0.041** |
| Species = Sp | 1 | 7002.00 | 4.44 | 0.280 |
| Pe x Si | 1 | 74.96 | 0.25 | 0.622 |
| Pe x Sp | 1 | 840.10 | 0.37 | 0.651 |
| **Si x Sp** | **1** | **1578.10** | **5.28** | **0.022** |
| **Pe x Si x Sp** | **1** | **2293.40** | **7.67** | **0.009** |
| Res | 60 | 298.84 |  |  |
| Total | 67 |  |  |  |
